# Editorial: Microbial co-cultures: a new era of synthetic biology and metabolic engineering, volume II

**DOI:** 10.3389/fmicb.2025.1587450

**Published:** 2025-03-20

**Authors:** Durgesh Kumar Jaiswal, Avinash Bapurao Ade, Tarun Belwal, Arthur Prudêncio De Araujo Pereira, Jay Prakash Verma

**Affiliations:** ^1^Department of Biotechnology, Graphic Era (Deemed to be University), Dehradun, India; ^2^Department of Botany, Savitribai Phule Pune University, Pune, Maharashtra, India; ^3^Texas A&M University, College Station, TX, United States; ^4^Federal University of Ceará, Soil Science Department, Soil Microbiology Laboratory, Fortaleza, Ceará, Brazil; ^5^Plant-Microbe Interaction Lab, Institute of Environment and Sustainable Development, Banaras Hindu University, Varanasi, Uttar Pradesh, India

**Keywords:** microbial consortia, metabolic engineering, industrial biotechnology, environment, synthetic consortia, sustainable agriculture, bio-economy

Microbial co-cultures—the controlled cultivation of two or more microbial species in a shared environment—have emerged as a transformative paradigm in synthetic biology and metabolic engineering, addressing limitations inherent in monoculture systems. By harnessing synergistic interactions, co-cultures enable modular division of labor, spatial organization, and cross-feeding dynamics that optimize metabolic pathways, enhance substrate conversion efficiency, and expand bioproduction capabilities (Brenner et al., 2008; Wang et al., 2024). This second volume of the Research Topic underscores the rapid advancements in this field, highlighting empirical successes in biomanufacturing, environmental remediation, and therapeutic development. It also emphasizes the critical role of computational modeling and systems biology in designing robust consortia, providing reassurance about the scientific rigor in the field (De Lorenzo et al., 2018).

A key advantage of co-cultures lies in their ability to compartmentalize complex biochemical tasks. For instance, *Saccharomyces cerevisiae* and *Clostridium autoethanogenum* co-cultures achieved a 40% increase in bioethanol yield compared to monocultures by segregating sugar fermentation and carbon fixation pathways, mitigating redox imbalances (Chen et al., 2024). Similarly, co-culturing *Escherichia coli* with *Pseudomonas putida* significantly altered their transcriptional profiles, particularly in central carbon metabolism, surface adhesion, and drug efflux pumps (Molina-Santiago et al., 2017). Such compartmentalization reduces metabolic burden, a persistent challenge in engineering single-strain systems for multi-step biosynthesis (Wang et al., 2024).

Co-cultures' application extends to pharmaceutical production, where incompatible biosynthetic pathways are partitioned between species. A notable example is the synthesis of the antimalarial precursor artemisinin-11,10-epoxide. By co-culturing *S. cerevisiae* (engineered for amorpha-4,11-diene production) with *Pichia pastoris* (expressing cytochrome P450 enzymes), titers reached 2.8 g/L—a 15-fold improvement over monoculture attempts (Ro et al., 2006). Similarly, co-cultures of Streptomyces *coelicolor* and *Bacillus subtilis* produced novel polyketide antibiotics via horizontal gene transfer, illustrating the potential for “synthetic ecology” in drug discovery (Traxler et al., 2013).

From an environmental perspective, microbial consortia are pivotal in sustainable bioprocessing. Lignocellulosic biomass degradation, a critical challenge in biofuel production, has been enhanced through fungal-bacterial synergy. A study demonstrated that co-culturing *Trichoderma reesei* and *Corynebacterium glutamicum* resulted in cellulose-to-glucose conversion efficiency. This strategy integrated fungal enzymatic hydrolysis with bacterial metabolic pathways to mitigate inhibitory byproducts, effectively overcoming critical challenges in lignocellulosic biomass valorization (Singh et al., 2022). This approach combined fungal enzymatic hydrolysis with bacterial metabolism of inhibitory byproducts, addressing key bottlenecks in lignocellulose valorization. Additionally, methane mitigation strategies employing *Methanotrophs* paired with *Alcanivorax* spp. Demonstrated a 63% reduction in atmospheric CH4 in landfill simulations, capitalizing on cross-species metabolite exchange (Chavan and Kumar, 2018). Despite the successes in microbial consortia, challenges persist in maintaining consortium stability, including competitive exclusion (where one species outcompetes others for resources), phage susceptibility, and metabolic cross-talk. Advances in dynamic regulation tools, such as quorum sensing-based feedback circuits, have improved population control. For instance, microbial consortia can be stabilized using strategies like multi-metabolite cross-feeding, which helps maintain stable population compositions by minimizing competition among strains and intermediate accumulation (Li et al., 2022). Machine learning has greatly improved our ability to predict microbial interactions. For example, researchers used models to forecast how bacteria interact based on their growth and metabolism. This helps engineer beneficial microbial communities by understanding how species interact, crucial for predicting community structure and function (Nestor et al., 2023).

This volume also highlights agricultural, environmental and industrial scalability considerations. The Research Topic features several noteworthy papers that contribute significantly to the field:

## Microbial interactions and defense mechanisms

Microbial interactions play a crucial role in the defense mechanisms of plants against various pathogens. Recent studies have highlighted the significance of these interactions, particularly in the context of enhancing agricultural resilience and sustainability.

Azzollini et al., studies, “*A Mass Spectrometry-Based Strategy for Investigating Volatile Molecular Interactions in Microbial Consortia: Unveiling a Fusarium-Specific Induction of an Antifungal Compound*,” explores volatile organic compounds (VOCs) involved in microbial interactions. Using advanced mass spectrometry, researchers analyzed VOC profiles produced by microbial consortia in response to Fusarium species, a significant plant pathogen responsible for widespread agricultural losses. A major finding was the identification of a novel antifungal compound, uniquely induced by *Fusarium* exposure. This compound exhibited potent antifungal activity, revealing a targeted microbial defense mechanism. The study highlighted that microbial communities dynamically adjust their metabolic output in response to specific pathogens, producing VOCs tailored to combat Fusarium. Comparative analysis showed distinct volatile profiles in Fusarium-challenged microbial consortia compared to controls, emphasizing the specificity of this interaction. The research provides valuable insights into how microbial communities use VOCs for communication and defense. These findings have significant implications for agriculture, particularly in developing eco-friendly antifungal agents to manage Fusarium-related crop diseases. By leveraging mass spectrometry-based approaches, the study lays a strong foundation for future investigations into microbial signaling and natural product discovery, advancing our understanding of microbial ecology and its practical applications.

Roylawar et al., investigated the impact of the root endophyte *Serendipita indica* on onion plants' resistance to the beet armyworm (*Spodoptera exigua*). The findings revealed that onions colonized by *S. indica* exhibited significantly reduced leaf damage from *S. exigua* larvae compared to non-colonized controls. Additionally, larvae feeding on *S. indica*-colonized plants showed decreased weight gain. Biochemical analyses indicated that this enhanced resistance is associated with increased activities of antioxidant and defense-related enzymes, including superoxide dismutase, peroxidase, polyphenol oxidase, and phenylalanine ammonia-lyase, as well as elevated hydrogen peroxide (H_2_O_2_) levels during the early stages of herbivory. Gene expression studies further confirmed the upregulation of defense-related genes in *S. indica*-colonized plants upon *S. exigua* attack. These results suggest that *S. indica* symbiosis primes onion plants for a more robust defense response against herbivorous insects, highlighting its potential as a biocontrol agent in sustainable agriculture.

Zhao et al., explored how antibiotic-resistant *Escherichia coli* strains can shield sensitive strains through cross-protection mechanisms. Utilizing an automated nanoliter droplet analyzer, they co-cultured β-lactamase-expressing (resistant) and non-expressing (sensitive) *E. coli* strains in the presence of the β-lactam antibiotic cefotaxime (CTX). The findings revealed a cross-protection window for the sensitive strain, extending up to approximately 100 times its minimal inhibitory concentration (MIC). Microscopic observations and enzyme activity assays indicated that antibiotic-induced bacterial filamentation contributes to this protective effect. Moreover, the study demonstrated that the extent of cross-protection is influenced by the disparity in β-lactamase activity between the co-cultured strains; larger differences shifted the cross-protection window toward higher CTX concentrations. These insights underscore the complexity of bacterial interactions under antibiotic stress and highlight the role of filamentation in enhancing the survival of sensitive bacteria. Understanding these dynamics is crucial for developing more effective antibiotic therapies and addressing challenges posed by bacterial resistance.

## Sustainable practices and environmental management

Sustainable practices and effective environmental management are critical in addressing the pressing challenges of pollution, resource depletion, and climate change. Recent research highlights innovative strategies that leverage microbial interactions and biotechnological advancements to foster sustainability.

Kherdekar and Ade, provides a comprehensive overview of integrated approaches for plastic waste management“ discusses the pressing issue of plastic pollution and evaluates various strategies to mitigate its environmental impact. The authors emphasize the importance of a multifaceted approach, combining mechanical recycling, chemical recycling, and biological degradation to address the complexity of plastic waste. They highlight that mechanical recycling is currently the most widely used method, accounting for approximately 20% of global plastic waste processing. However, its effectiveness is limited by the degradation of plastic properties after multiple recycling cycles. In contrast, chemical recycling offers the advantage of breaking down plastics into their monomers, allowing for the production of virgin-quality polymers. Despite its potential, chemical recycling is energy-intensive and currently accounts for < 1% of plastic waste treatment. The article also explores biological degradation, noting that certain microorganisms have been identified with the capability to decompose specific types of plastics, such as polyethylene terephthalate (PET). While promising, this approach is still in the experimental stage and faces challenges related to scalability and efficiency. The authors advocate for an integrated waste management system that leverages the strengths of each method, coupled with policy interventions and public awareness campaigns, to effectively tackle the growing plastic waste crisis.

Irsad et al., addresses the critical challenge of global food security amid an increasing population. Traditional reliance on synthetic pesticides has led to issues such as environmental contamination, pest resistance, and health hazards. The authors advocate for eco-friendly alternatives, highlighting entomopathogens (ENMs) as promising biopesticides. ENMs, including bacteria and fungi, possess unique properties: bacteria are host-specific, while fungi have a broader host range, effectively targeting both soil-dwelling and terrestrial insect pests. The study emphasizes the need for identifying, isolating, and formulating these microbial agents to overcome current pest management challenges. The authors conclude that integrating ENMs into pest management strategies can sustain productivity, enhance environmental health, reduce pesticide reliance, and conserve natural resources.

## Metabolic engineering and secondary metabolite production

Lei et al., investigated the role of the sco1842 gene, designated as ccr1 (combined-culture related regulatory protein no. 1), in the production of secondary metabolites (SMs) in *Streptomyces coelicolor* A3(2). The study utilized combined-culture techniques involving *Tsukamurella pulmonis* TP-B0596 to stimulate SM production. Mutational analysis revealed that disruption of ccr1 led to a significant reduction in major SMs, including undecylprodigiosin (RED). Further examination showed that Ccr1 homologs are highly conserved across the *Streptomyces* genome. Although Ccr1 lacks traditional conserved motifs, detailed analysis identified a helix–turn–helix (HTH) motif in the N-terminal region and a helicase C-terminal domain (HCTD) motif in the C-terminal region in some homologs. RNA sequencing indicated that Ccr1 functions as a nucleoid-associated protein (NAP), influencing genome-wide transcription. Additionally, RT-qPCR demonstrated that ccr1 expression is upregulated in combined-culture with *T. pulmonis*, suggesting its involvement in bacterial interactions. In *Streptomyces nigrescens* HEK616, knocking out the ccr1 homolog resulted in decreased production of streptoaminals and 5aTHQs. These findings suggest that Ccr1 and its homologs play a pivotal role in regulating SM production in *Streptomyces* species, particularly in response to interactions with other bacteria.

As we advance into this new era of synthetic biology and metabolic engineering, the integration of microbial co-cultures will likely play a pivotal role in addressing global challenges such as food security, environmental sustainability, and health care. The findings presented in this Research Topic underscore the importance of interdisciplinary research and collaboration among microbiologists, engineers, and biotechnologists. In conclusion, “*Microbial Co-cultures: A New Era of Synthetic Biology and Metabolic Engineering, Volume II*” serves as a critical resource that not only highlights current advancements but also paves the way for future innovations in microbial biotechnology. The collective insights from this Research Topic are essential for researchers aiming to harness the power of microbial interactions to drive sustainable solutions across various industries.
